# Efficacy of ProTaper Retreatment System in Root Canals Obturated with Gutta-Percha Using Two Different Sealers and GuttaFlow

**DOI:** 10.1155/2011/676128

**Published:** 2011-10-23

**Authors:** Jaya Siotia, Shashi Rashmi Acharya, Sunil Kumar Gupta

**Affiliations:** ^1^Department of Conservative Dentistry and Endodontics, Faculty of Dentistry, Melaka Manipal Medical College, Manipal University, Manipal, Karnataka 576104, India; ^2^Department of Conservative Dentistry and Endodontics, Manipal College of Dental Sciences, Manipal University, Manipal, Karnataka 576104, India

## Abstract

*Objective*. To compare the efficacy of ProTaper retreatment files in removing three different obturating materials. *Study Design*. Forty-five human, single-rooted premolars were divided into three experimental groups. Group 1 was obturated with gutta-percha and AH Plus sealer, Group 2 was obturated with gutta-percha and zinc oxide eugenol sealer, and Group 3 was obturated with GuttaFlow. Retreatment was done using the ProTaper universal rotary retreatment files. Root halves were visualized using magnifying loops at 3X magnification and optical stereomicroscope at 10X magnification. Images were analyzed using AutoCAD 2004 software to calculate area of the remaining debris in the canal. For statistical analysis were used variance test and ANOVA. *Results*. Total debris/canal area ratio between the three groups showed a statistically significant difference (*P* < 0.001). *Conclusion*. ProTaper retreatment system did not produce completely clean canals in any of the groups. However, it had the best efficacy towards removing silicon based obturating material GuttaFlow.

## 1. Introduction

Endodontic failure occurs even when the highest standard and the most meticulous treatment procedure is adhered to. When conventional root canal treatment fails, endodontic retreatment is the preferred option as it is one of the most conservative methods. 

The main goal of retreatment is to regain access to the apical foramen by complete removal of the root canal filling material. Biomaterial-centered biofilm form in root canal obturating material in failed endodontic cases [1] and necrotic tissue and bacteria, covered by obturating material, may be responsible for periapical inflammation [2]. Therefore, as much as possible, the obturating material must be removed to reduce the number of microorganisms within the canal. 

Removal of obturating material can be achieved by several methods such as ultrasonic technique, chemical methods, and heat pluggers [3–5]. Nickel-titanium rotary instruments have also been used [6, 7].

Recently, ProTaper universal retreatment files (Densply Tulsa Dental) have been introduced which are designed specifically to remove obturating material from root canals.

Many obturating materials, techniques, and sealers have been developed. Lateral compaction of gutta-percha is a commonly used method for obturation and is regarded as a reference when considering other obturation techniques. GuttaFlow (Coltène/Whaledent, Langenau, Germany), a new obturating material, is a combination of Gutta-percha in powder form and polydimethylsiloxane based sealer.

There is limited information about the removability of this new root canal filling material for retreatment purpose and also few studies have investigated the effectiveness of the new ProTaper universal retreatment instruments in the removal of obturating material during endodontic retreatment. Therefore, the aim of this study was to compare the efficacy of ProTaper retreatment files in the removal of root canal fillings obturated with gutta-percha and AH Plus sealer, gutta-percha and Zinc oxide Eugenol sealer, and GuttaFlow.

## 2. Materials and Methods

### 2.1. Specimen Preparation

Forty-five extracted human single-rooted, single-canal premolars with completely formed apices were selected for the study. Soft tissue and calculus were mechanically removed from the root surfaces using ultrasonic scalers. Teeth were autoclaved and stored in 0.2% thymol. They were then decoronated using diamond disk (Diatech Swiss Dental, Switzerland) to obtain root segments of 13 mm length.

### 2.2. Root Canal Preparation and Obturation 

Working length was determined by inserting a size 10 K file (Dentsply Maillefer) into the root canal until it was visible at the apical foramen and subtracting 1 mm from that length. Root canal preparation was done using ProTaper universal rotary files (Dentsply Maillefer, Ballaigues, Switzerland) as per manufacturer's instructions. All canals were prepared upto F2 ProTaper file. Irrigation was done after each instrument with 10 mL of 2.5% NaOCl. When instrumentation of the root canals was completed, 17% ethylenediaminetetraacetic acid was used for 1 minute for smear layer removal, and the canals were again irrigated with 5 mL of 2.5% NaOCl. Canals were then dried with paper point. Teeth were randomly divided into three experimental groups. 

Group 1 was obturated with gutta-percha and AH Plus sealer using lateral compaction technique. Group 2 was obturated with gutta-percha and zinc oxide eugenol sealer using lateral compaction technique. Group 3 was obturated with GuttaFlow.

Groups 1 and 2 canals were obturated with 0.02/25 gutta-percha master cone (Dentsply, Petro'polis, RJ, Brazil), accessory cones, and sealer (group 1: AH Plus (Dentsply Detrey, Konstanz, Germany), group 2: zinc oxide eugenol (Endofill; Dentsply), using Lateral compaction technique. For group 3, GuttaFlow (Coltène/Whaledent, Langenau, Germany) was manipulated and inserted into the canals as per manufacturer's instructions. The access cavities were sealed with glass ionomer cement (GC Corporation, Tokyo, Japan).

Teeth were radiographed in buccolingual and mesiodistal direction to confirm the adequacy of root fillings. The samples were stored at a temperature of 37°C and 100% humidity for 2 weeks.

### 2.3. Root Canal Retreatment

Root fillings were removed using the ProTaper universal NiTi rotary retreatment files (Tulsa Dental, Tulsa, OK) as per manufacturer's instructions. Canals were instrumented in a crown-down sequence using ProTaper D1 file (0.09/0.30 mm) to remove filling material from the coronal third of the canals. Middle and apical thirds of the canals were instrumented using ProTaper D2 (0.08/0.25 mm) and ProTaper D3 (0.07/0.20 mm) files, respectively, in a brushing action. 5 mL of 2.5% NaOCl was used after each instrument followed by a final rinse of 5 mL saline. Further canal refinement was not done unlike previous studies [8, 9] because the purpose of this experiment was to assess solely the efficacy of ProTaper retreatment system alone in removing the root canal fillings.

Retreatment was deemed complete when the last file reached the working length, there was no filling material covering the instrument, and canal walls appeared smooth and free of debris. During retreatment, all instruments were used in 3 canals and were then discarded.

### 2.4. Analysis of Filling Debris

The roots were grooved longitudinally in the buccolingual direction with a diamond disk (Diatech Swiss Dental, Switzerland) and split into halves with a chisel. The two halves were visualized using magnifying loops (ErgonoptiX, Netherlands) at 3X magnification. The half with a greater amount of filling debris was then taken for examination under an optical stereomicroscope (Olympus SZ-11, Japan) at 10 X magnification.

**Figure 1:**
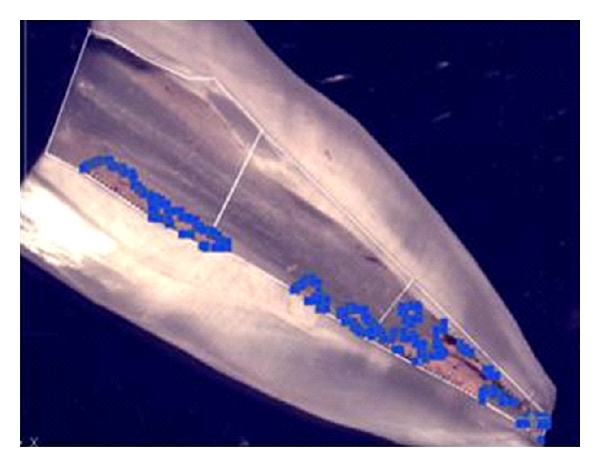
Outline of the total canal area and the filling debris area using AutoCAD 2004 software tool.

Images were captured with a digital camera coupled to the stereomicroscope and analyzed using AutoCAD 2004 software (Mechanical Desktop Power Pack; Microsoft, Redmond, Wash, USA). Canal walls and filling debris were identified based on the difference in the color. A single operator used the software tool to outline the canal area and the filling debris area in each third (cervical, middle, and apical), as well as the total canal area (Figure 1).

The filling debris/canal area ratios were considered as a unit of analysis and expressed as percentage of filling material left after reinstrumentation.

The analysis was carried out in SPSS 16 using repeated measures analysis of variance and ANOVA. A *P* value of 0.05 was considered to be statistically significant. 

First, the canal thirds (apical, middle, and coronal) within each group were compared. Second, intergroup comparison was done within each canal third. Repeated measures analysis of variance was used for this part of statistical analysis. Finally, intergroup comparison using ANOVA considered the total canal area to calculate the filling debris/canal area ratio.

## 3. Results

All three groups used in the study had some filling material left within the root canal after reinstrumentation with ProTaper retreatment files. The maximum percentage of remaining debris was in group 1 followed by group 2. The least amount of remaining debris was in Group 3. In Group 1, the middle and apical third of the canal had maximum amount of debris remaining, whereas in Groups 2 and 3, only the apical third had maximum remaining debris (Table 1).

However, when the debris ratio was compared at each third (coronal, middle and apical) across the three groups, there was no statistically significant difference (*P* = 0.272). There was a statistically significant difference in the total debris/canal area ratio between Group 1 and Group 3 followed by Group 1 and Group 2 (*P* < 0.001) (Table 2).

## 4. Discussion

Endodontic nonsurgical retreatment is a comprehensive field with its own science, literature, specific technologies, best materials, and escalating range of techniques that are, at times, required to achieve clinical success [10]. Complete removal of preexisting filling material from canals is a prerequisite for successful nonsurgical root canal retreatment. 

Different techniques have been used to evaluate the remaining filling material: radiographs [11], clearing techniques and digitized images [12], operating microscopes [13], and scanning electron microscopy (SEM) [14]. Residual gutta-percha and sealer have been measured using evaluation scales, for example, mild, moderate, and severe [15]. More recently micro-CT has been used [16].

In the current study, an optical stereomicroscope was used to visualize the remaining filling material. The roots were visualized using magnifying loops at 3X magnification before selecting the half for stereomicroscopic analysis. This gave a clearer picture of the remaining debris as compared to naked eye visualization and helped in selecting the appropriate root half for further analysis. The AutoCAD 2004 software gave the exact area of the remaining debris in the root canal. This method is more precise as compared to the evaluation scale which is a subjective procedure and is bound to have subjective errors.

All three obturating materials selected for the study are gutta-percha based. The difference is in the types of sealer used. Group 1 is an epoxy-amide-based sealer, Group 2 is zinc oxide eugenol-based sealer and Group 3 contained a silicone-based sealer, polydimethylsiloxane.

In the current study, all groups had some amount of remaining debris. This is in accordance to previous studies in which completely clean canal walls were not produced by any of the techniques investigated [9, 17, 18]. 

In Groups 2 and 3, the apical third had a mean percentage of remaining filling material greater than the middle and the cervical third. This is due to increased anatomical variability and difficulty of instrumentation of the apical third. Moreover, the master apical file size, F2, has a tip diameter of 0.25 mm, whereas the tip diameter of D3 file, used to clean the apical portion of the root canal, is 0.20 mm. D3 file tip did not bind to the canal walls and permitted a complete cleaning action. This indicates that further root canal filing with files of larger diameter is necessary to completely remove the obturating material from the apical part of the root canal.

In Group 1, both the middle and apical thirds had significant amount of remaining debris. This observation is consistent with that of Zmener et al. and Kosti et al. [6, 15]. Epoxy resin-based sealers adhere better to the dentin walls, making their removal with rotary instruments difficult. The middle third has greater compaction of obturating material and sealer. Moreover, greater sealer penetration into the dentinal tubule at the middle third could be the reason for greater amount of remaining debris in the middle third. Studies have shown that the depth of penetration of root canal sealers into dentinal tubules using the lateral compaction technique is influenced by the root canal level, with penetration decreasing apically [19]. The reason for the significant amount of apical debris in this group is the same as that explained for Groups 2 and 3.

Protaper retreatment files showed better cleanliness in the cervical third. A Similar finding was observed by Bramante et al. who related it to the dental anatomy in this region and speed of rotary instruments [20]. 

Group1 had significantly greater total debris as compared to Groups 2 and 3. The sealers used have different constituents and adhesive behavior; therefore, it is not surprising that varying amounts of materials remained. Zinc oxide eugenol-based sealer has less adhesion to the root canal wall than a resin-based sealer [21, 22]. No information is yet available on the adhesion of silicone-based sealers to dentine, however, GuttaFlow does not exhibit chemical bonding to the canal wall. RoekoSeal, which is considered as the initial form of GuttaFlow, was removed more easily from the canals than a resin-based sealer [15]. Obturation done using lateral compaction technique tends to result in better condensation of obturating material [23], and this type of obturation is more difficult to remove as compared to the cold flowable GuttaFlow.

## 5. Conclusions

Within the experimental conditions of the present study, it can be concluded that, (1) Protaper universal retreatment files did not produce completely clean canals in any of the groups; (2) among the materials tested, it showed the best efficacy towards removing silicone-based obturating material GuttaFlow. (3) Gutta-percha with epoxy resin-based sealer left maximum amount of debris in the canal. 

A Large amount of debris remains adhered to the wall after removal of obturating material. ProTaper retreatment files alone are not sufficient for removing obturating material. Therefore, effective refiling of the canal is important to obtain cleaner root canals during retreatment. Further research is necessary towards standardizing the canal morphology and individually checking the efficacy of ProTaper retreatment files in different types of canals.

## Figures and Tables

**Table 1:** Mean (SD) filling debris area/canal area ratio obtained in the coronal, middle and apical third and also in the total canal.

Groups	Coronal third	Middle third	Apical third	Total canal
G1	0.35 (0.18)	0.59 (0.20)	0.55 (0.45)	0.45 (0.16)
G2	0.24 (0.14)	0.26 (0.24)	0.39 (0.22)	0.27 (0.13)
G3	0.11 (0.05)	0.21 (0.19)	0.38 (0.28)	0.18 (0.08)

**Table 2:** Mean difference in the debris ratio of the total canal between the three groups.

Group (i)	Group (j)	Mean difference	95% confidence interval		*P* value
1	2	.1837529	0.066	0.30	.001*
2	3	−.0847673	0.03	−0.20	.197
1	3	−.2685202	0.15	0.38	.000*

*Significant at 5% level.
